# The FGFR4 Homolog KIN-9 Regulates Lifespan and Stress Responses in *Caenorhabditis elegans*

**DOI:** 10.3389/fragi.2022.866861

**Published:** 2022-05-20

**Authors:** Avijit Mallick, Leo Xu, Sakshi Mehta, Shane K. B. Taylor, Hannah Hosein, Bhagwati P. Gupta

**Affiliations:** Department of Biology, McMaster University, Hamilton, ON, Canada

**Keywords:** *kin-9*, FGFR4, *pry-1*, *miR-246*, aging, stress response, ER-UPR, Fgf signaling

## Abstract

Fibroblast growth factor receptors (FGFRs) regulate diverse biological processes in eukaryotes. The nematode *Caenorhabditis elegans* is a good animal model for studying the roles of FGFR signaling and its mechanism of regulation. In this study, we report that KIN-9 is an FGFR homolog in *C. elegans* that plays essential roles in aging and stress response maintenance. *kin-9* was discovered as a target of *miR-246*, a microRNA that is positively regulated by the Axin family member *pry-1*. We found that animals lacking *kin-9* function were long-lived and resistant to chemically induced stress. Furthermore, they showed a reduced expression of endoplasmic reticulum unfolded protein response (ER-UPR) pathway genes, suggesting that *kin-9* is required to maintain a normal ER-UPR. The analysis of GFP reporter-based expression in transgenic animals revealed that KIN-9 is localized in the intestine. Overall, our findings demonstrate that *kin-9* is regulated by *miR-246* and may function downstream of *pry-1*. This study prompts future investigations to understand the mechanism of miRNA-mediated FGFR function in maintaining aging and stress response processes.

## Introduction

Aging is the gradual deterioration of cellular and tissue functions that are regulated by both genetic and environmental factors (Kenyon, 2010; Lapierre and Hansen, 2012; Uno and Nishida, 2016). Genetic factors include components of conserved signaling pathways that regulate a host of cellular processes that are crucial for the development and proper functioning of healthy adults. Thus, it is essential to identify and understand the function of pathway components so that they can be targeted not only to promote normal growth, but also to develop potential treatments for associated diseases. To this end, our group is investigating the function of an Axin scaffolding protein homolog in the nematode *Caenorhabditis elegans*, PRY-1, which is necessary for the formation of many tissues and cell types, as well as for maintaining stress responses and aging (Mallick et al., 2019b, 2020, 2021a; Mallick and Gupta, 2022). To gain insights into the genetic network of *pry-1,* we performed mRNA and miRNA transcriptome profiling experiments (Ranawade et al., 2018; Mallick et al., 2019a). Together with biochemical and genetic studies, these data identified many factors that interact with *pry-1*, including *aak-2/AMPK*, *daf-16/FOXO*, and *crtc-1/CRTCs* (Mallick et al., 2020; 2021b). Our miRNA transcriptome analysis revealed six differentially expressed miRNAs in *pry-1* mutants, five of which (*lin-4*, *miR-237*, *miR-48*, *miR-84*, and *miR-241*) were upregulated, and one (*miR-246*) was downregulated (Mallick et al., 2019a). miRNAs are non-coding RNAs that regulate target gene expression by binding to their conserved 3ʹ-untranslated region (UTR) or, less commonly, to the 5ʹ-UTR and coding sequences (Stefani and Slack, 2008; Ambros and Ruvkun, 2018; O’Brien et al., 2018). Studies have shown that miRNAs regulate diverse biological processes (Ambros and Ruvkun, 2018; O’Brien et al., 2018).

The present study focuses on *miR-246* and one of its targets in mediating aging and stress resistance. *miR-246* was reported to be the highest-fold upregulated gene during aging in both wild-type and long-lived *daf-2* (insulin/insulin-like growth factor-1 signaling (IIS) receptor homolog) mutant (De Lencastre et al., 2010; Pincus et al., 2011). However, the mechanism underlying the action of *miR-246* remains unclear. The results described here suggest that *miR-246* acts genetically downstream of *pry-1* to regulate the expression of a fibroblast growth factor receptor (FGFR) homolog to promote longevity and stress resistance in animals.

FGF signaling is conserved in eukaryotes and plays an important role in development and disease (DeVore et al., 1995; Ornitz and Itoh, 2015; Xie et al., 2020). Studies in mammalian models have revealed that the pathway is regulated by miRNAs (Yin et al., 2015; Copeland and Simoes-Costa, 2020); however, detailed mechanisms of interactions, specific processes, and pathway components are currently lacking. In this regard, the *C. elegans* system holds significant potential because it contains conserved miRNA families and FGF signaling that can be targeted by forward and reverse genetic approaches. While EGL-17 (FGF) and EGL-15 (FGFR) were previously shown to be necessary for sex specific muscle development (DeVore et al., 1995), they are not essential for the lifespan and stress responses in worms. Here, we provide evidence that KIN-9 is an FGFR4 family member that is repressed by *mir-246*. The receptor tyrosine kinase (RTK) domain of KIN-9 shows a high degree of sequence and structural similarity to those of FGFR4 mammalian family members. In agreement with this, we found that the KIN-9 overexpression phenotype resembled that of activated FGF signaling in *C. elegans*. A phenotypic analysis revealed that while *kin-9* mutants are long-lived and stress-resistant, *miR-246* mutants show the opposite phenotypes. We also found that *kin-9* RNAi fully suppressed the lifespan and stress sensitivity of the *miR-246* mutants. To further validate the regulatory relationship between *miR-246* and *kin-9*, a chimeric GFP-*kin-9*-3ʹ UTR reporter was used, which showed increased fluorescence in *miR-246* mutant animals. These data, together with the upregulation of KIN-9 in both the *miR-246* and *pry-1* mutants, support a model where *pry-1* positively regulates *miR-246*, which in turn inhibits *kin-9* expression. Analysis of *kin-9::GFP* transgenic animals revealed that the gene is expressed in the pharynx and intestine. Its presence in the intestine supports the role of *kin-9* in lifespan maintenance, similar to that described for many other long-lived mutant strains (An and Blackwell, 2003; Libina et al., 2003; Taylor and Dillin, 2013). As *kin-9* mutants are resistant to stress, we examined the expression of unfolded protein response (UPR) pathway components and chaperones. The results showed that the expression of the endoplasmic reticulum (ER) UPR components were downregulated, suggesting that lower *kin-9* activity is beneficial for protein homeostasis. Overall, the results described in this study demonstrate the essential role of *kin-9* in regulating *mir-246*-mediated lifespan and stress response in *C. elegans*.

## Materials and Methods

Parts of the methods section are in the Supplementary Data S1.

### Strains and Culture Conditions

Worms were cultured on standard NGM plates using established protocols and *Escherichia coli* strain OP50 as a food source (Brenner, 1974). The strains were maintained at 20°C unless otherwise mentioned.

### RNAi

RNAi-mediated gene silencing was performed using a protocol previously published by our laboratory (Mallick et al., 2021a). Plates were seeded with *Escherichia coli* HT115 expressing either dsRNA specific to candidate genes or empty vector (L4440). Synchronized gravid adults were bleached, and eggs were plated. After becoming young adults, animals were analyzed for stress sensitivity and lifespan (Mallick et al., 2021a).

### Computational Analysis

To identify the targets of *miR-246*, the computational algorithms TargetScan (Jan et al., 2011), PicTar (Lall et al., 2006), PITA (Kertesz et al., 2007) and STarMirDB (Rennie et al., 2016) were used. These tools predict miRNA targets based on 3ʹ UTR seed matches of genes. The searches generated *miR-246* target candidates (Supplementary Figure S1; Supplementary Table S2).

### Lifespan Analysis

Lifespan experiments were done following adult-specific RNAi treatment using a previously described protocol (Mallick et al., 2020). Animals were grown on NGM OP50 seeded plates till the late L4 stage after which they were transferred to RNAi plates. For lifespan analysis at different temperatures, animals were grown till the late L4 stage at 20°C following which they were shifted to either 15°C or 25°C. Plates were then screened daily for dead animals and surviving worms were transferred every other day till the progeny production ceased. Censoring was done for animals that either escaped, burrowed into the medium, showed a bursting of intestine from the vulva, or underwent bagging of worms (larvae hatch inside the worm and the mother dies).

### U0126 Inhibitor Assay

NGM plates with U0126 inhibitor (U120-1 MG, Sigma-Aldrich) of 30 µM was adapted from described previously (Reiner et al., 2008; Sharanya et al., 2015). A stock concentration of 10 mM U0126 was prepared in DMSO and was added to plates right before pouring to achieve a plate concentration of 30 µM. Synchronized L1 animals were plated and allowed to grow till young adult stage following which stress sensitivity to 200 mM was examined.

### Oil Red O Staining

Neutral lipid staining was done on synchronized day-1 adult animals using Oil Red O dye (Thermo Fisher Scientific, United States) following the previously published protocol. Quantification was then done using ImageJ software as described previously (Mallick and Gupta, 2020).

## Results

We previously reported that PRY-1 regulates the expression of a set of heterochronic miRNAs, including the *lin-4* and *let-7* family members (Mallick et al., 2019a). *miR-246*, which affects the stress response and aging-related processes (De Lencastre et al., 2010), was also discovered in that study. It was found that *miR-246* is not involved in the *pry-1*-mediated heterochronic pathway. Furthermore, its expression was downregulated in *pry-1(mu38)* larvae and adults (Mallick et al., 2019a).

### *kin-9* Expression is Regulated by *miR-246* and its 3ʹ UTR Contains miRNA Consensus Binding Sites

**FIGURE 1:**
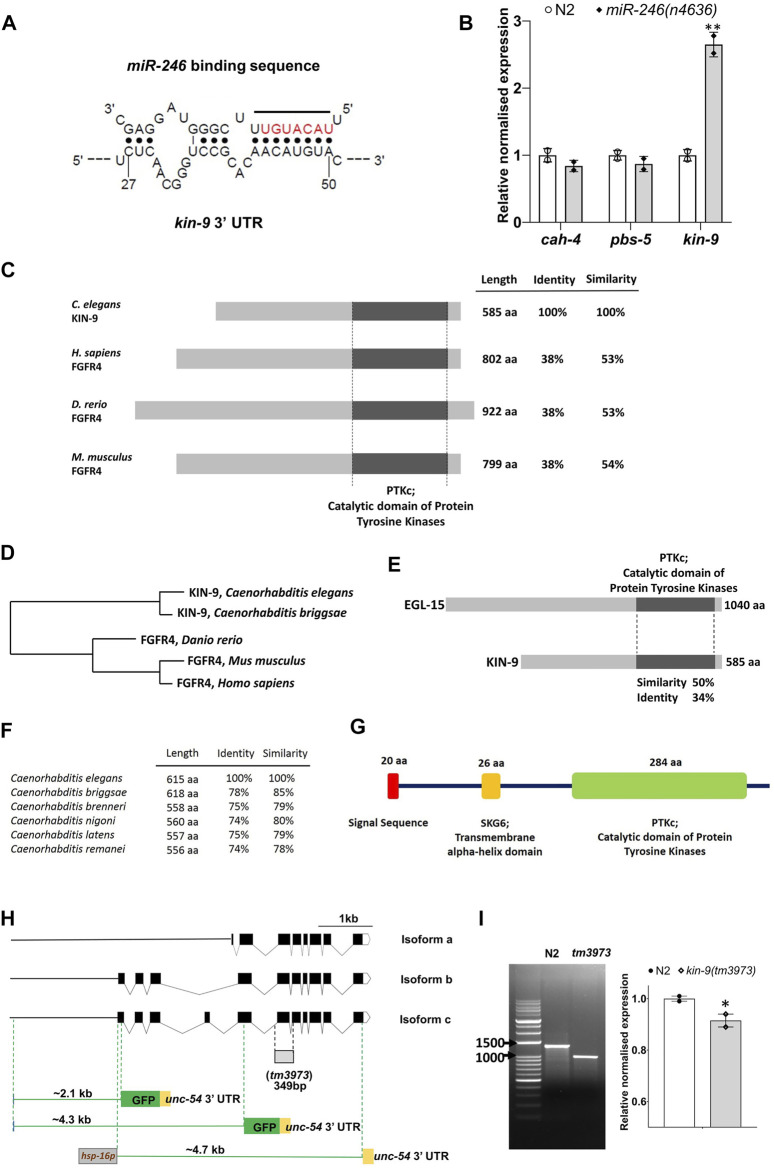
*miR-246* mutants show overexpression of *kin-9*. **(A)** The predicted binding site of *miR-246* at the 3′ UTR of *kin-9* mRNA. **(B)** Expression analysis of three candidate genes *cah-4, pbs-5* and *kin-9* in the *miR-246* mutants. **(C)** Schematic representation of KIN-9 and FGFR4 proteins from *Caenorhabditis elegans*, *Homo sapiens, Danio rerio*, and *Mus musculus* with percent identity and similarity indicated relative to *C. elegans* KIN-9. Conserved domains are aligned and are depicted with sizes, all presented to scale. **(D)** Phylogenetic tree of proteins shown in panel (C). **(E)** Schematic representation of *C. elegans* KIN-9 and EGL-15 proteins with percent identity and similarity indicated. **(F)** Similarities and identities between the KIN-9 proteins in the *Caenorhabditis* genus. **(G)** Protein domains and structure of KIN-9 protein. **(H)** Schematic dendrograms showing all the isoforms and tm3973 deletion allele of *kin-9* with exons (black solid boxes), introns (bent lines) and upstream sequences (solid straight line). Regions used for creating transcriptional reporters and heat shock promoter-driven *kin-9* overexpression are also shown. **(I)** PCR and qPCR analyses of the *tm3973* allele. Gel image showing the shorter fragment of *kin-9* transcript and bar graph showing *kin-9* mRNA levels in the *tm3973* mutants. **(B, I)** Each data represents the mean of two replicates and error bars the standard error of means. Significance was calculated using Bio-Rad software (one-way ANOVA) and significant differences are indicated by stars (*): * (*p* < 0.05), ** (*p* < 0.01).

To understand the biological role of *miR-246*, we focused on identifying its target genes. Because the phenotype caused by a miRNA deletion is expected to result from the increased activity of its target(s), we hypothesized that *miR-246* loss-induced short lifespan and enhanced stress sensitivity would be suppressed by depletion of its target gene activity. The predicted targets of *miR-246* were identified using computational algorithms (see Methods). We assessed the transcript levels of top three potential targets (*cah-4*, *kin-9*, and *pbs-5*) in an *miR-246* deletion mutant that lacks the entire transcript and 5ʹ upstream sequence (*n4636:* 518 bp length) (Miska et al., 2007) (Figures 1A,B). It was expected that the transcript level of the target gene would be higher than normal in the miRNA mutant background. Our results revealed that, while *kin-9* expression was significantly upregulated compared to the control, there was no change in *cah-4* and *pbs-5* expression (Figure 1B; Supplementary Figure S2).

Sequence analysis revealed that KIN-9 is a member of the FGFR4 family (http://www.wormbase.org). The RTK domain of KIN-9 is approximately 53% similar between the mouse and human FGFR4 proteins (Figures 1C,D). Outside this domain, no significant sequence similarity with FGFR4 was detected (Figure 1C). We also used the secondary structure prediction tool Jpred4 (http://www.compbio.dundee.ac.uk/jpred/) (Drozdetskiy et al., 2015), which confirmed KIN-9 homology with human FGFR4 (PDB ID: 6jpj, 6jpe, 5jkg, 4uxq, 4qrc, and 4qqt). Finally, to determine whether the KIN-9 RTK domain possesses conserved tyrosine kinase phosphorylation sites present in FGFR, the online program group-based prediction system (GPS 5) was utilized (http://gps.biocuckoo.cn/online.php) (Xue et al., 2011). The analysis revealed six such sites that, together with secondary structure prediction, established KIN-9 as a bona fide FGFR family member in *C. elegans* (Supplementary Figure S3). It is worth mentioning that a previously characterized FGFR in *C. elegans*, EGL-15, shares 50% sequence similarity with the RTK domain of KIN-9 (Figure 1E; Supplementary Figure S4) (Schutzman et al., 2001). KIN-9 orthologs were also found in other nematode species (http://www.wormbase.org) (Figures 1F). The *kin-9* gene is predicted to produce three isoforms of varied sizes (478 aa (a), 585 aa (b), and 615 aa (c), all of which have a conserved RTK domain. The longest isoforms (b and c) also possess an N-terminal signal sequence and a transmembrane alpha-helix domain (Figures 1G,H).

### *kin-9* mutants are Stress Resistant and Long-Lived Whereas *hsp::kin-9* Animals are Stress Sensitive and Die Prematurely

A deletion mutant of *kin-9*, *tm3973*, was used to investigate the gene function. The mutation was found to remove a portion of the RTK domain, leading to multiple in-frame stop codons (see Supplementary Data S1). These changes are expected to cause truncated proteins for all three isoforms (167 aa, 271 aa, and 304 aa) (Figure 1H). Interestingly, a cDNA analysis showed the presence of a roughly 990 bp transcript in *kin-9* deletion mutant (*tm3973*) worms, indicating a readthrough transcription (Figure 1I). While it is unclear whether the *tm3973* mutation allows translation, any products arising from this allele are expected to be non-functional.

**FIGURE 2:**
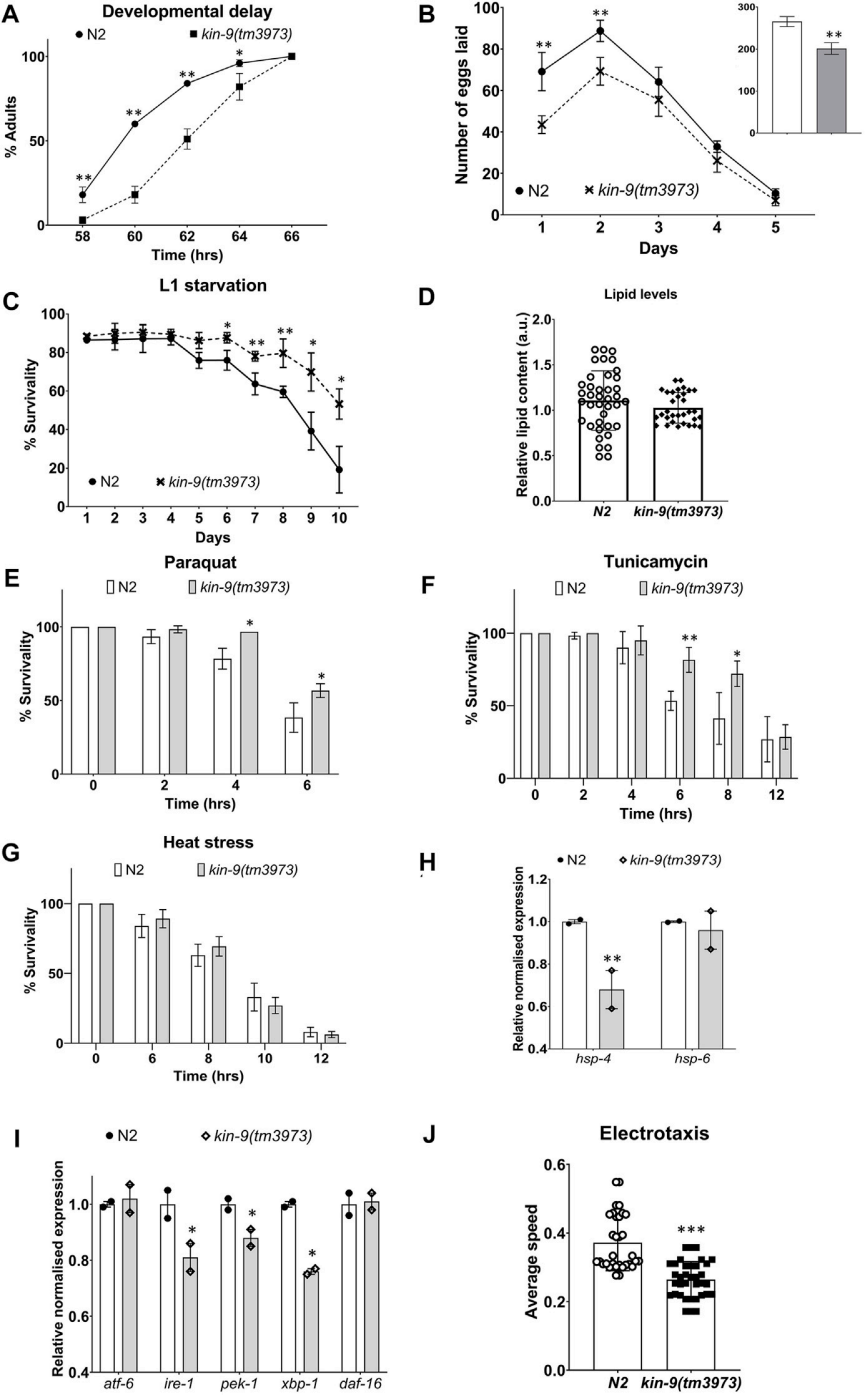
*kin-9* mutants exhibit resistance to heat and chemical-induced stress. **(A)** Graph showing the time taken to reach adulthood. **(B)** Line graph showing the number of eggs laid by *kin-9* mutants compared to N2. Smaller bar graph showing the total number of eggs laid (N2- clear bar; *kin-9(tm3973)* grey bar). **(C)** Survival graph of *kin-9(tm3973)* L1 worms upon starvation compared to N2. **(D)** Quantification of neutral lipids using Oil Red O staining. **(E,F)** Bar graph showing the survivability of *kin-9* mutants compared to N2 following paraquat (200 mM) and tunicamycin (25 ng/μL) over a period of 6 and 12hrs respectively. **(G)** Survivability of *kin-9* mutants compared to N2 at 35°C over a period of 12hrs. **(H,I)** qPCR analysis of daf-16 genes in *kin-9(tm3973)* adults compared to N2. Data in **(H–I)** represent the mean of two replicates and error bars the standard error of means. Significance was calculated using Bio-Rad software (one-way ANOVA). **(J)** Electrotaxis analysis of day-1 *kin-9(tm3973)* adults. **(A–G,I)** Data represent the mean of two replicates (n > 40 animals in each replicate) and error bars represent the standard deviation. Statistical analyses were done using multiple unpaired *t*-tests and significant differences are indicated by stars (*): * (*p* < 0.05), ** (*p* < 0.01), *** (*p* < 0.001).

*kin-9* deletion mutant (*tm3973*) animals exhibited no obvious morphological defects but appeared to have a slight growth delay and lay significantly fewer eggs (Figures 2A,B); in addition, L1 larvae showed resistance to starvation (Figure 2C). Since brood size and L1 survival may be affected by lipid levels (Watts and Ristow, 2017; Ranawade et al., 2018), we performed Oil Red O staining and found no change in neutral lipid levels (Figure 2D). The *tm3973* animals also showed increased resistance to paraquat and tunicamycin but were insensitive to heat stress (Figures 2E–G). These data suggest that *kin-9* plays an important role in the stress response maintenance. Further support for this conclusion comes from the expression analysis of the endoplasmic reticulum unfolded protein response (ER-UPR) genes. We found that *kin-9* mutants affected the ER-UPR pathway, as evidenced by the reduced expression of *ire-1*, *pek-1*, *xbp-1*, and the chaperone *hsp-4* (Figures 2H,I). Consistent with these results, the mutants exhibited electrotaxis defects associated with chronic stress (Figure 2J) (Taylor et al., 2021). No change in the expression of mitochondrial UPR components was detected in the absence of *kin-9* function (Figures 2H,I).

**FIGURE 3:**
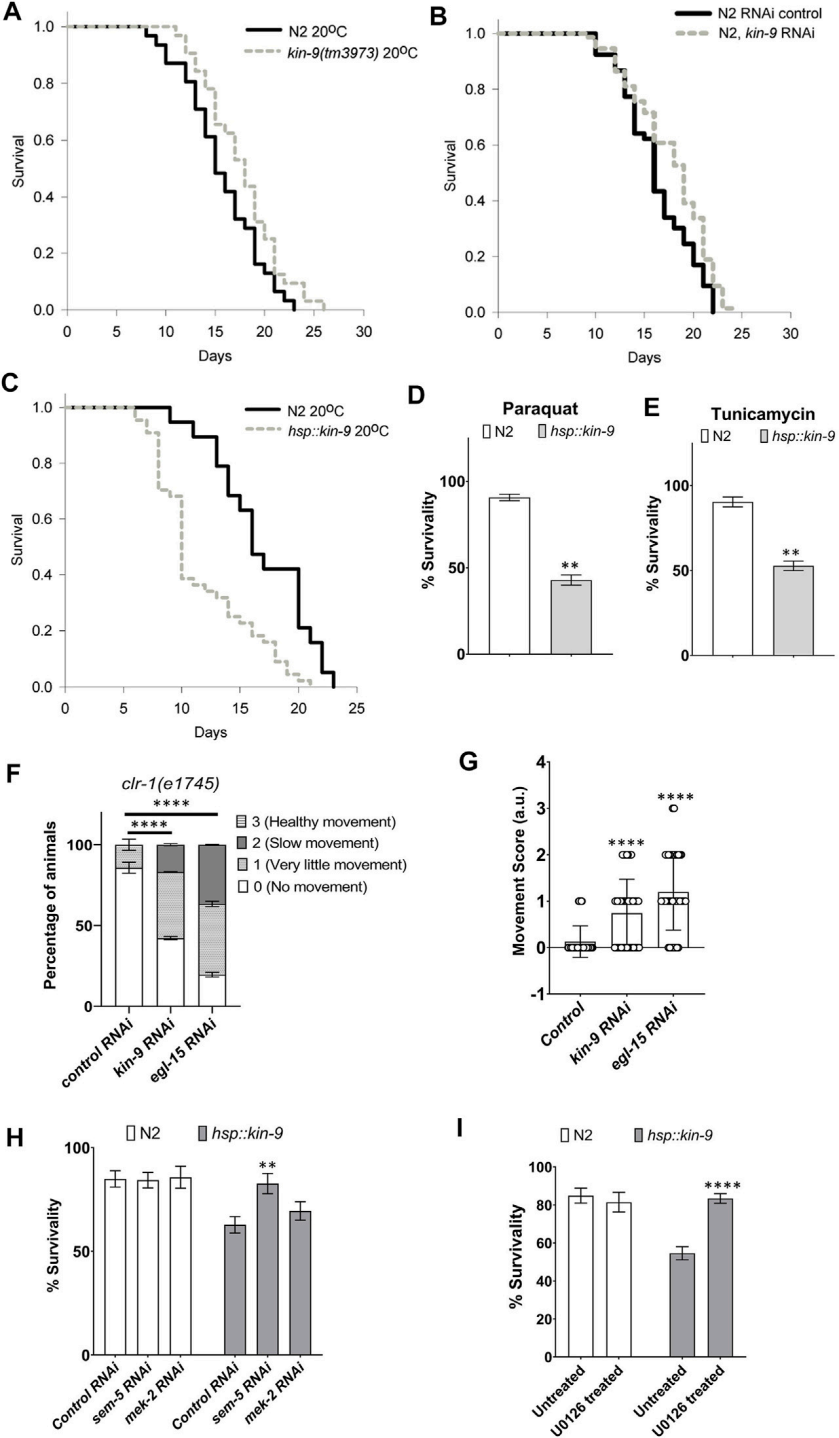
Loss of *kin-9* function extends lifespan. **(A)** Lifespan graphs of *kin-9 (tm3973)* mutants compared to N2 **(B)** Lifespan analysis of animals following control and *kin-9* RNAi knockdown during adulthood. **(C)** Lifespan graphs of *hsp::kin-9* animals compared to N2. **(A–C)** See the Methods section and Supplementary Table S3 for statistics performed. **(D,E)** Bar graph showing the survivability of *hsp::kin-9* adults compared to N2 following paraquat (200 mM) and tunicamycin (25 ng/μL) exposure for 2hrs. **(F,G)** Staggered bar graphs **(F)** and bar graphs **(G)** showing the analyses of movement defect in *clr-1* mutants following control, *kin-9* and *egl-15* RNAi. In panel F movement is quantified on a scale of 0–3 and in panel G average score for movement has been plotted. **(H)** Bar graphs showing the survivability of N2 and *hsp::kin-9* adults in 200 mM paraquat for 2hrs following control, *sem-5* and *mek-2* RNAi knockdown. **(I)** Bar graphs showing the survivability of N2 and *hsp::kin-9* adults in 200 mM paraquat for 2hrs following U0126 inhibitor treatment. **(D–I)** Data represent the mean of two to three replicates (*n* > 50 animals in each replicate) and error bars represent the standard deviation. Statistical analyses were done using multiple unpaired *t*-tests with Welch correction **(D,E,H,I)**, one-way ANOVA with multiple comparison test **(G)** and 2-way ANOVA with Sidak multiple comparison test **(F)**. Significant differences are indicated by stars (*): ** (*p* < 0.01), *** (*p* < 0.001) and **** (*p* < 0.001).

Because *miR-246* mutants are short-lived (De Lencastre et al., 2010), we examined the lifespan phenotype of animals lacking *kin-9* function. Consistent with *kin-9* being a downstream target, mutant animals showed an extended lifespan (Figure 3A; Supplementary Table S3). Similar lifespan changes were observed in the RNAi-treated animals (Figure 3B; Supplementary Table S3). The mutants exhibited slightly increased body bending rates but no change in pharyngeal pumping (Supplementary Figure S5).

If the absence of *kin-9* results in a lifespan extension, then its increased levels should give rise to the opposite phenotype. To test this hypothesis, *hsp::kin-9* transgenic lines were generated that overexpressed *kin-9* following heat treatment (see Supplementary Data S1). High levels of *kin-9* expression in these animals were confirmed using qPCR (Supplementary Figure S6). Consistent with the longer lifespan of *kin-9* mutants, transgenic animals overexpressing *kin-9* were small, short-lived, and exhibited slower pharyngeal pumping and body bending (Figure 3C; Supplementary Figures S5, S7, S8). The aging phenotype was similar at other growth temperatures (15 and 25°C, Supplementary Figure S7; Supplementary Table S3). Moreover, *hsp::kin-9* animals exhibited an increased stress sensitivity to both paraquat and tunicamycin (Figures 3D,E). Altogether, these data demonstrate an important role for *kin-9* in regulating the normal lifespan and stress responses of animals.

Interestingly, we found that *hsp::kin-9* animals were unusually transparent when grown at 25°C (Supplementary Figure S8), an appearance that resembled ‘Clear (Clr)’ phenotype reported earlier in *C. elegans* that have activated FGF signaling (Supplementary Figure S8) (Borland et al., 2001). To investigate whether *kin-9* genetically interacts with *clr-1*, we performed RNAi experiments. The results showed that while *kin-9* RNAi had no effect on the Clr phenotype, it suppressed movement defects of animals (Figures 3F,G). In control experiments, *egl-15* knockdown suppressed both the Clr and movement defects of *clr-1* mutants (Figures 3F,G).

Additionally, we performed RNAi knockdowns of two of the FGF pathway components, MEK-2 (Erk Kinase) and SEM-5 (Grb2) (Borland et al., 2001). The results showed that *sem-5* RNAi suppressed stress sensitivity of *hsp::kin-9* animals (Figure 3H). No such response was not observed in the case of *mek-2* (Figure 3H). Since *C. elegans* contains two Erk kinases (*mek-1* and *mek-2*), we reasoned that there may be a genetic redundancy and, therefore, used a MEK inhibitor U0126 (Favata et al., 1998; Reiner et al., 2008; Sharanya et al., 2015). As expected, treating *hsp::kin-9* animals with U0126 fully rescued their stress sensitivity to PQ (Figure 3I). Altogether, these data support our conclusion that KIN-9 function is mediated by the ERK kinase signaling, consistent with its role in regulating FGF signaling.

### *kin-9* is Expressed in the Pharynx, Intestine, and Tail Region

**FIGURE 4:**
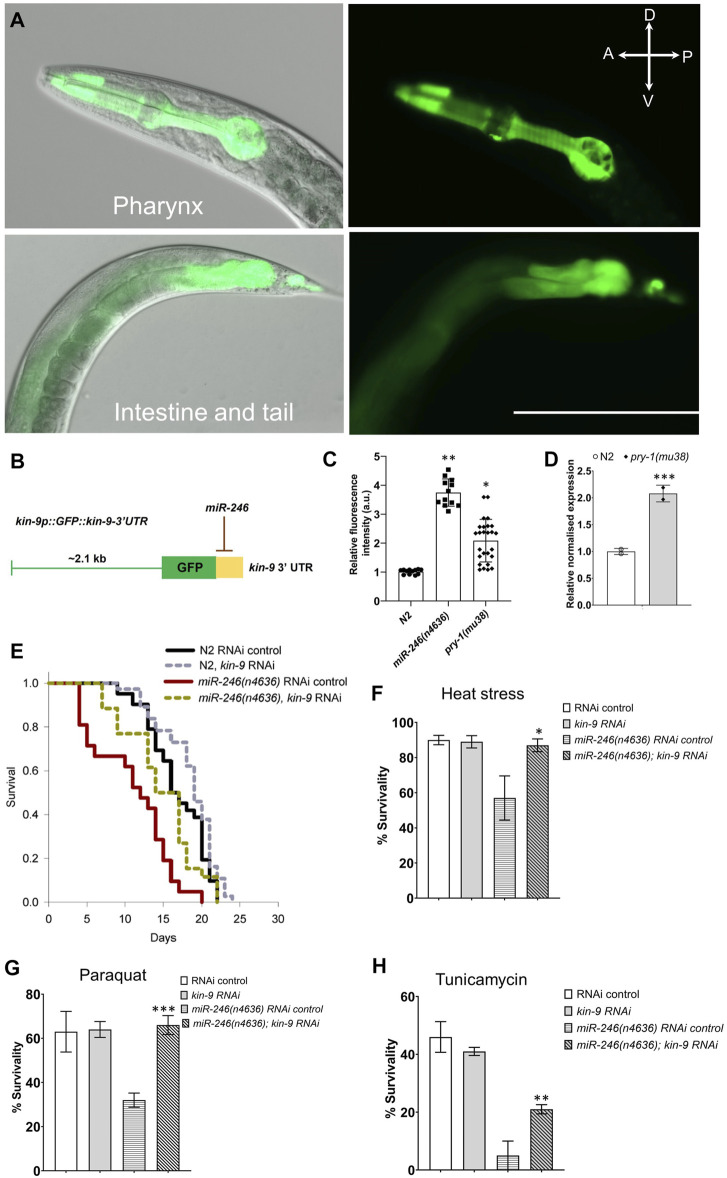
Lowering *kin-9* activity suppresses *miR-246* mutant defects. **(A)** *kin-9p (2.1 kb):GFP* analysis shows expression in the pharynx, intestine, and tail neurons. **(B)** Schematic diagram of a *kin-9p::GFP::kin-9* 3′ UTR construct showing potential *miR-246* binding at the 3′ UTR of the kin-9 mRNA transcript. **(C)** Bar graph showing the GFP analysis using the array *kin-9p::GFP::kin-9* 3′ UTR in *miR-246 (n4636)* and *pry-1(mu38)* adults compared to control. Data represent the mean of two replicates (*n* > 25 animals in each replicate) and error bars represent the standard deviation. Statistical analyses were done using one-way ANOVA with Dunnett’s post hoc test and significant differences are indicated by stars (*): * (*p* < 0.05), ** (*p* < 0.01). **(D)** qPCR analysis of *kin-9* gene in the *pry-1(mu38)* adults compared to control. Data represent the mean of two replicates and error bars the standard error of means. Significance was calculated using Bio-Rad software (one-way ANOVA) and significant differences are indicated by stars (*): *** (*p* < 0.001). **(E)** Lifespan analysis of N2 and *miR-246 (n4636)* animals following control and *kin-9* RNAi knockdown. See the Supplementary Data S1 and Supplementary Table S3 for statistics performed. **(F–H)** Bar graphs showing survivability of N2 and *miR-246 (n4636)* animals following control and *kin-9* RNAi when exposed to heat stress (35°C), paraquat (200 mM for 2 h) and tunicamycin (25 ng/μl for 2 h). Data represent the mean of two replicates (*n* > 50 animals in each replicate) and error bars represent the standard deviation. Statistical analyses were done using one-way ANOVA with Dunnett’s post hoc test and significant differences are indicated by stars (*): * (*p* < 0.05), ** (*p* < 0.01).

Considering the *kin-9’s* essential role in *C. elegans*, we wanted to identify the cells and tissues in which the gene is expressed. To this end, transgenic strains carrying *kin-9::GFP* reporters were generated. Two different constructs were utilized (see Supplementary Data S1), the longest of which (4.3 kb) contained a part of an exon that is common to all isoforms, whereas the shorter one (2.1 kb) is specific to isoforms b and c (Figure 1H). The analysis of transgenic lines showed GFP fluorescence throughout their lifespan, which agrees well with previously published transcriptomic data (Golden et al., 2008; Grün et al., 2014). We found that *kin-9p(2.1 kb)::GFP* adults exhibited fluorescence in the pharynx, intestine, and certain cells located in the tail (Figure 4A). While *kin-9p(4.3 kb)::GFP* animals exhibited a similar pattern, interestingly, no fluorescence was observed in the posterior region (Supplementary Figure S8). It is possible that the longer fragment has certain inhibitory sequences that contribute to differences in expression. More experiments involving the dissection of regulatory sequences are needed to investigate this possibility. The intestinal localization of KIN-9 and its persistence during adulthood agree well with the expression of other genes that promote lifespan and stress response maintenance (An and Blackwell, 2003; Libina et al., 2003; Taylor and Dillin, 2013). However, the specific role of KIN-9 in the pharynx remains to be determined.

### *kin-9* 3ʹ UTR is Targeted by *miR-246* and *kin-9* RNAi Rescues *miR-246(n4636)* Defects

Since miRNAs function mainly by binding to the 3ʹ UTR of target genes (Ambros and Ruvkun, 2018; O’Brien et al., 2018), we examined whether *miR-246* affects the transcriptional regulation of *kin-9*. To this end, transgenic lines were generated containing a chimera of GFP and *kin-9* 3ʹ UTR under the control of the *kin-9* promoter (*kin-9p(2.1 kb)::GFP::kin-9 UTR*) (Supplementary Data S1; Figure 4B). As expected, GFP fluorescence increased approximately four-fold when the construct was introduced into *miR-246(n4636)* animals (Figure 4C). Since *miR-246* is positively regulated by *pry-1* (Mallick et al., 2019a), we also examined *kin-9p(2.1 kb)::GFP::kin-9UTR* fluorescence in *pry-1* mutants and found similar upregulation (Figure 4C). Consistent with this, qPCR analysis revealed that *kin-9* transcript levels were high in animals lacking the *pry-1* function (Figure 4D). Altogether, these data show that *pry-1* and *miR-246* negatively regulate *kin-9* expression.

As *kin-9* expression is upregulated in *miR-246* mutants and *miR-246* regulates *kin-9* transcript levels, we tested whether *miR-246(n4636)* phenotypes could be rescued by reducing *kin-9* function. The lifespan and stress sensitivity defects of *miR-246* mutants were significantly rescued by *kin-9* RNAi (Figures 4E–H; Supplementary Figure S9, Supplementary Table S3). Together with transcript analysis and data from previous sections, these results demonstrate that *kin-9* is involved in lifespan regulation and acts downstream of *miR-246*. Interestingly, no phenotypic rescue was observed by knocking down *kin-9* in the *pry-1* mutant animals (Supplementary Figure S10, Supplementary Table S3), suggesting that *kin-9* is not the sole effector of *pry-1* function in aging-related processes. These findings are consistent with the interaction of *pry-1* with multiple pathway components to regulate aging and the stress response in *C. elegans*.

## Discussion

We identified *kin-9* as a new target of the miRNA *miR-246* in *C. elegans* and demonstrated its essential function in regulating stress responses and the lifespan of animals. Sequence analysis of *kin-9* revealed that it is a member of the FGFR family, with the RTK domain being the most similar to FGFR4. Furthermore, its overexpression using a *kin-9* transgenic system resulted in a Clr phenotype similar to that observed in activated FGF signaling conditions (Borland et al., 2001; Schutzman et al., 2001). The Clr phenotype is characterized by the accumulation of clear fluid within the pseudocoelomic cavity. Such animals appear to have floating intestines with fluid-filled body cavities and are short, immobile, and sterile (Borland et al., 2001; Schutzman et al., 2001). A similar trait was also observed in other FGF pathway component mutants (Schutzman et al., 2001). Interestingly, *kin-9* RNAi did not affect the Clr phenotype of *clr-1* mutants. However, movement defect of animals was rescued similar to *egl-15* knockdown. Furthermore, we found that the MEK inhibitor U0126 fully suppressed the stress sensitivity of animals overexpressing *kin-9*. Overall, these data lead us to conclude that *kin-9* regulates FGF signaling in *C. elegans*.

Studies in other animal models have reported the miRNA-mediated regulation of FGFs and FGFRs. For example, *miR-140* regulates *FGF9* during lung development, and *miR-200a*, *miR-20a*, and *miR-217* regulate *FGF4*, *FGF13*, and *FGFR12*, respectively, during the establishment of the neural crest territory (Yin et al., 2015; Copeland and Simoes-Costa, 2020). The work described here represents the first such study in *C. elegans* and provides a unique opportunity to investigate the miRNA-mediated regulation of FGFR. It is worth mentioning that, while the mammalian systems contain twenty-three FGF family members (FGF1-23) and four FGFRs (FGFR1-4), *C. elegans* carries only two ligands (EGL-17 and LET-756) and two receptors (EGL-15 and KIN-9) (Borland et al., 2001; Ornitz and Itoh, 2015; Xie et al., 2020). The simplicity of the worm system, together with its short lifespan and powerful genetic approaches to manipulate the genes, make it possible to investigate the mechanism of *kin-9* function and identify other pathway components that act genetically downstream of *miR-246*.

Previously, *miR-246* was identified in a transcriptomic study in our lab as being positively regulated by *pry-1* (Mallick et al., 2019a). Considering that miRNAs are necessary for lifespan maintenance (De Lencastre et al., 2010), we aimed to identify their targets to further understand the role of the *pry-1-miR-246* genetic network. While our *in-silico* analysis revealed three genes with consensus *miR-246* binding sites in their 3ʹ UTR, *kin-9* was the only gene with increased expression in the *miR-246* mutant background. Consistent with this, a *GFP* transgene containing the *kin-9* 3ʹ UTR was responsive to *miR-246* activity, that is, in the absence of *miR-246*, fluorescence was significantly increased.

Analysis of *kin-9* mutants has revealed the essential role of this gene in regulating stress responses and aging-related processes. While animals lacking *kin-9* function showed resistance to stress treatments, reduced expression of heat shock chaperone and ER-UPR genes, and a longer lifespan, transgenic lines overexpressing *kin-9* showed the opposite phenotypes. These data show that perturbations in *kin-9* function have opposite effects on stress response and ER-UPR gene expression. Studies have shown that *hsp-4* expression levels do not always correlate with stress sensitivity of animals. Thus, for example, *hsp-4* is downregulated in both *atf-6* (stress resistant) and *xbp-1* (stress sensitive) mutants (Taylor and Dillin, 2013; Burkewitz et al., 2020). More work is needed to fully elucidate the cellular and molecular mechanisms of *kin-9* in ER-UPR and stress response maintenance. It may be that a reduction in *hsp-4* and other ER-UPR genes in *kin-9* mutants is associated with beneficial effects such as improved proteostasis, thereby contributing to increased stress resistance and better health of animals. In this regard, experiments examining *kin-9*’s involvement in protein aggregation, various forms of stress, and degradation and clearing of intracellular factors should provide valuable information.

Consistent with the lifespan phenotype of *kin-9* mutants, the gene is expressed in the intestine, a tissue known to be the primary player in nutrient uptake and metabolic activities (Libina et al., 2003; Rera et al., 2013). Studies on aging have shown that the intestine communicates with other parts of the body, such as neurons and muscles, leading to the activation of downstream effectors. For example, the IIS transcription factor DAF-16, which functions mainly in neurons and the intestine, affects muscle health and mitochondrial mass, suggesting cross-talk between these tissues (Libina et al., 2003; Uno and Nishida, 2016; Wang et al., 2019; Mallick et al., 2020; Mallick and Gupta 2022). Thus, it is conceivable that *kin-9* regulates stress responses and lifespan by maintaining a healthy gut, which in turn signals to other tissues to promote their health.

Our data support a model in which *pry-1* promotes *miR-246* expression which in turn inhibits *kin-9* expression. However, the precise mechanism underlying this genetic relationship remains unclear. Although *kin-9* expression was inhibited by *pry-1*, knockdown of the gene did not suppress the *pry-1* phenotype. It may be that *kin-9* regulates only a small set of *pry-1* downstream components and is unable to alter *pry-1* signaling significantly in an independent manner. Previous studies on *pry-1/Axin* have revealed its genetic network, which includes multiple signaling components (Mallick et al., 2019b). For example, PRY-1 interacts with AAK-2/AMPK in the muscle in a cell-non-autonomous manner to regulate DAF-16 expression in the intestine to promote the lifespan and muscle health of animals (Mallick et al., 2020). PRY-1 also regulates the CABIN1 domain-containing protein, PICD-1, to affect calcineurin signaling and CRTC-1-dependent transcription (Mallick et al., 2021b). Recently, we identified several genes (*cpz-1/CTSZ*, *cdk-1/CDK1*, *rnr-1/RRM1*, *his-7/H2AX*, and *ard-1/HSD17B10*) that function downstream of *pry-1* to regulate the lifespan and stress response of animals (Mallick et al., 2021a). Taken together, these findings demonstrate that *pry-1* acts as a master regulator of aging-related processes and functions by coordinating the activities of diverse genes and pathways.

KIN-9 is the first identified FGFR family member in *C. elegans* that plays an essential role in aging and stress response maintenance. Previously, FGF signaling (EGL-15-EGL-17) was reported to be necessary to promote similar processes mediated by the homologs of the transmembrane proteins Klotho (KLO-1 and KLO-2), although EGL-15 and EGL-17 mutants independently do not have any such phenotypes (Château et al., 2010). Studies in higher eukaryotes have also shown that Klotho promotes lifespan and functions as a co-receptor for FGFRs (Kuro-o et al., 1999; Ornitz and Itoh, 2015). Moreover, there is evidence that mammalian FGFs cause age-related changes. For example, age-associated impairment of human mesenchyme-derived progenitor cells can be reversed by FGF2 treatment (Hurley et al., 2016). Additionally, the activated FGF2 pathway causes increased fat accumulation in aged human skeletal muscles (Mathes et al., 2021).

Our analysis of *kin-9* as a target of *miR-246* and its potential role genetically downstream of *pry-1* prompt future studies to investigate the mechanism of its regulation and conservation across eukaryotes. Whether *kin-9* utilizes the known components of FGF signaling remains to be determined. Interestingly, *kin-9* RNAi was shown earlier to delay the development of *let-60/Ras* mutant animals (Byrne et al., 2007). The identification of *kin-9* pathway components and their interactions with *pry-1* holds significant promise in advancing our understanding of Axin signaling in stress maintenance and aging.

## Data Availability

The original contributions presented in the study are included in the article/Supplementary Materials, further inquiries can be directed to the corresponding author.
