# Supplementary figures and images for: The FGFR4 Homolog KIN-9 Regulates Lifespan and Stress Responses in Caenorhabditis elegans
Source: Front Aging. 2022 May 20;3:866861. doi: 10.3389/fragi.2022.866861 (PMC9261393; doi:10.3389/fragi.2022.866861)

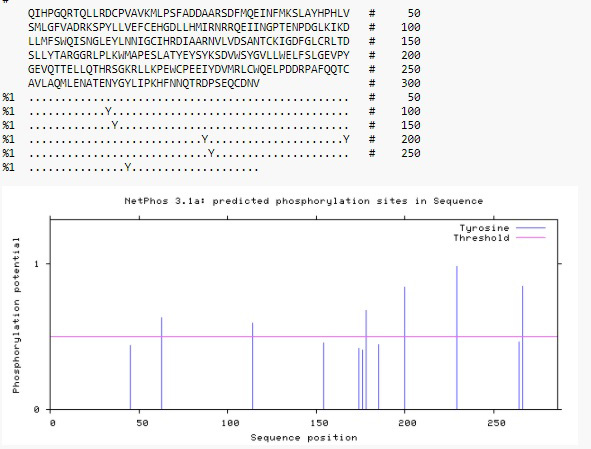

Supplement: Supplementary file 2 [file Image3.JPEG]

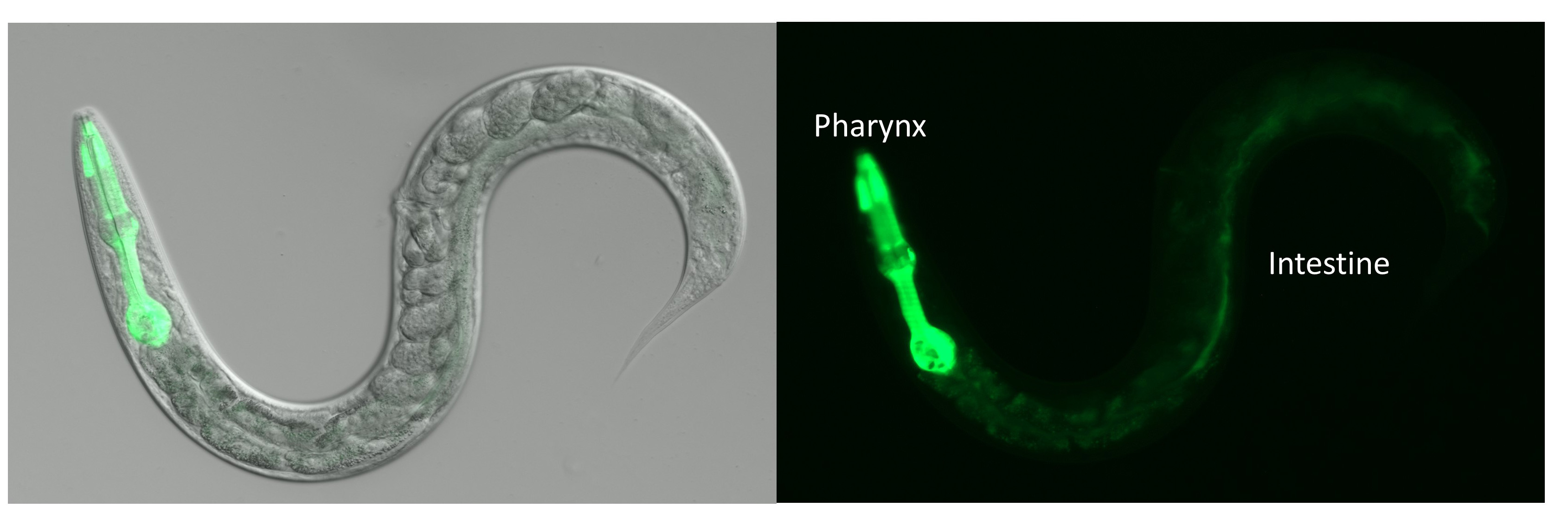

Supplement: Supplementary file 3 [file Image9.JPEG]

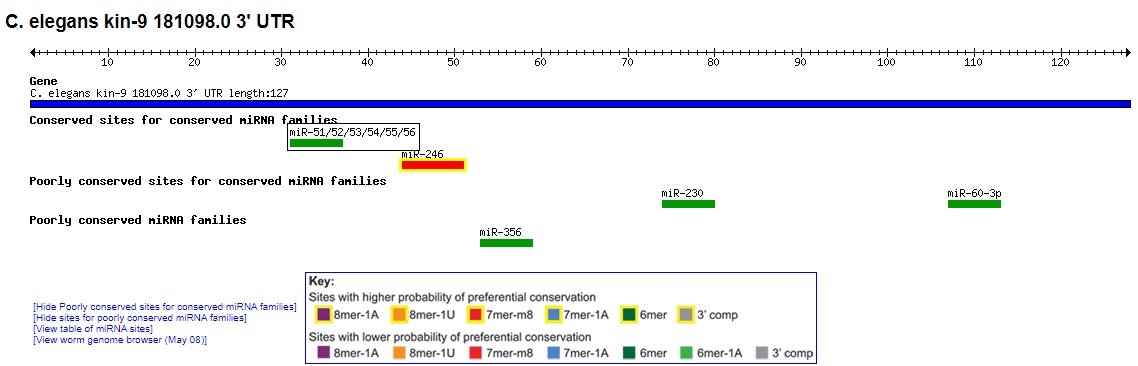

Supplement: Supplementary file 4 [file Image1.JPEG]

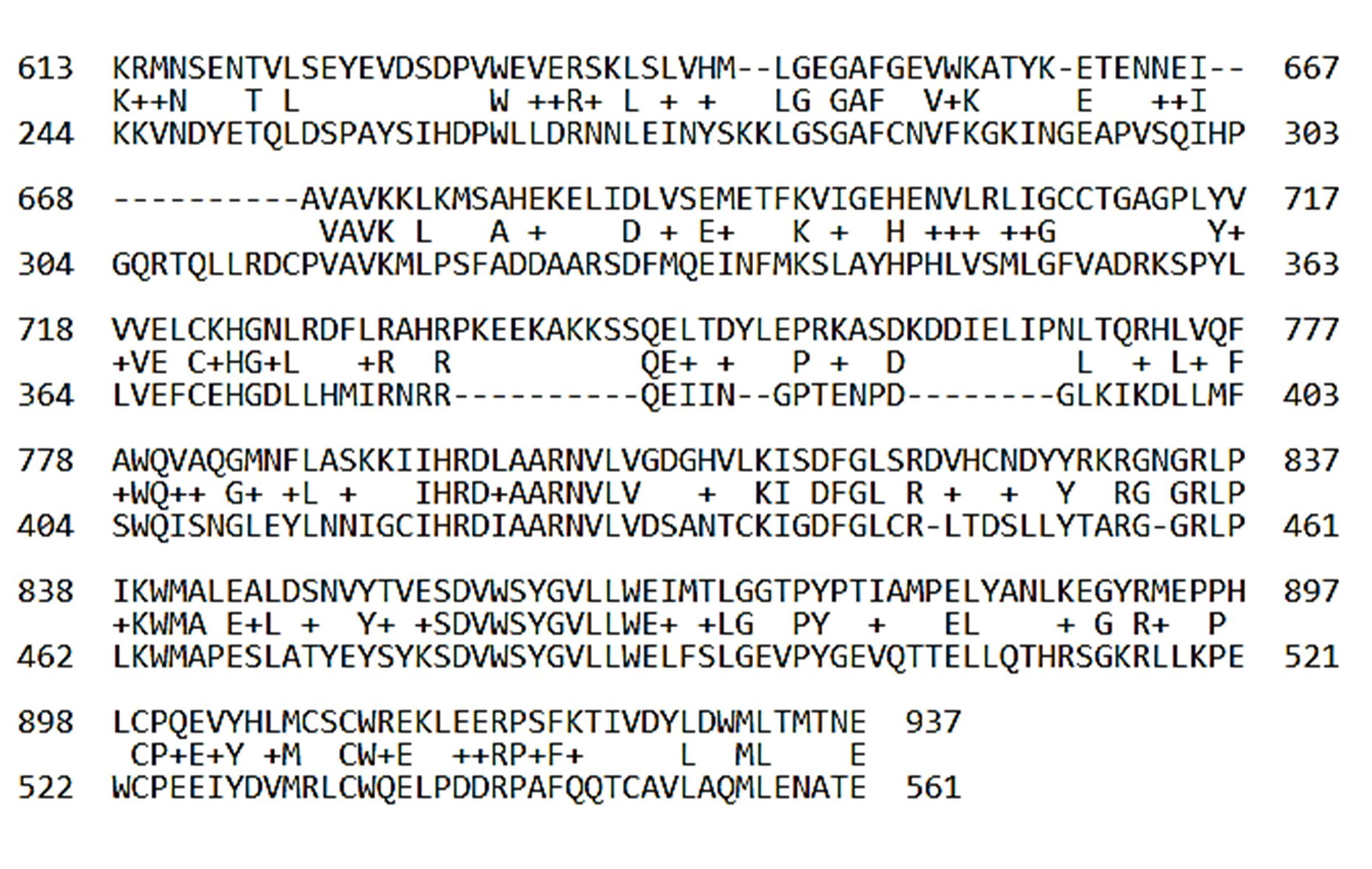

Supplement: Supplementary file 5 [file Image4.JPEG]

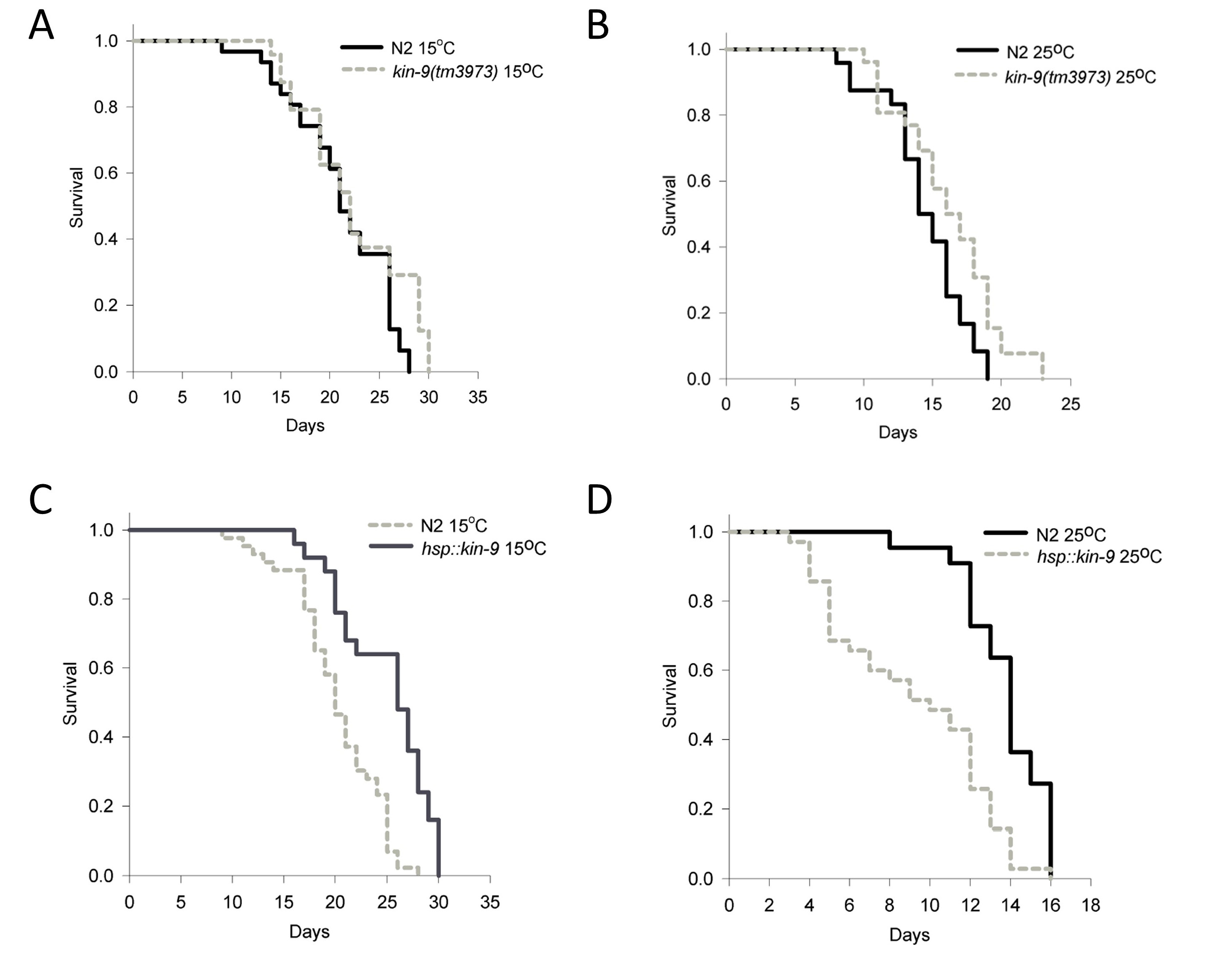

Supplement: Supplementary file 6 [file Image7.JPEG]

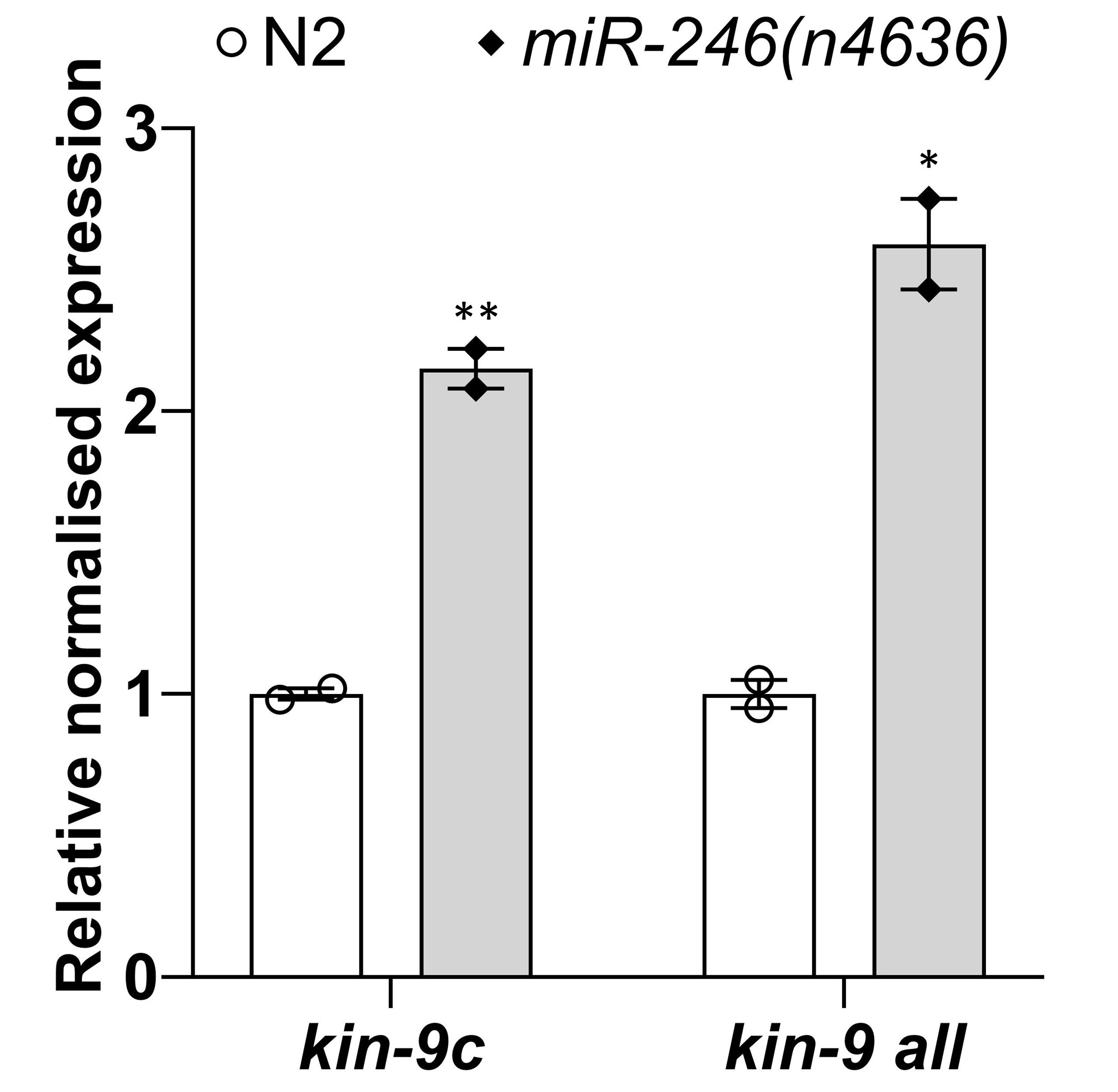

Supplement: Supplementary file 7 [file Image2.JPEG]

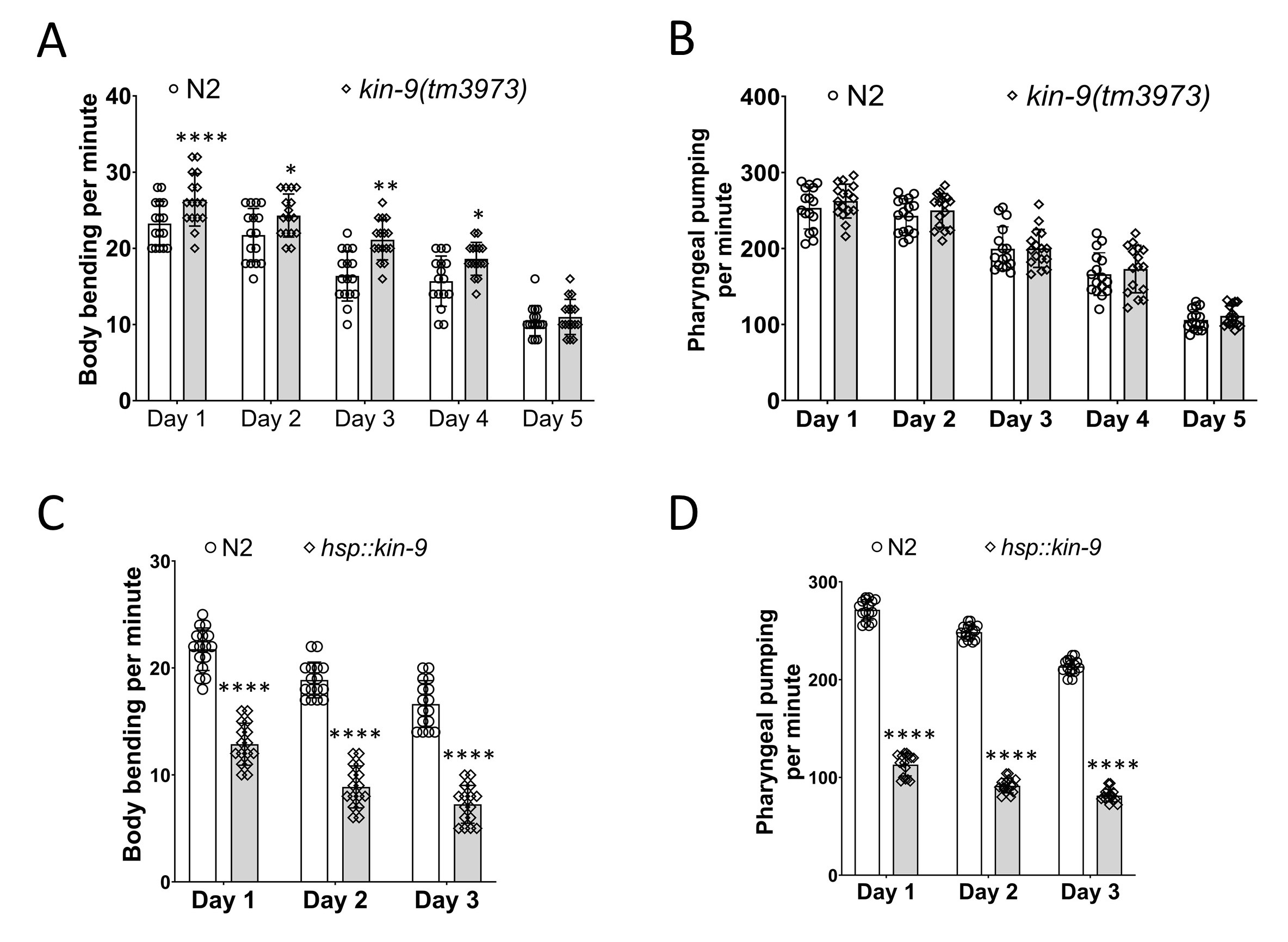

Supplement: Supplementary file 8 [file Image5.JPEG]

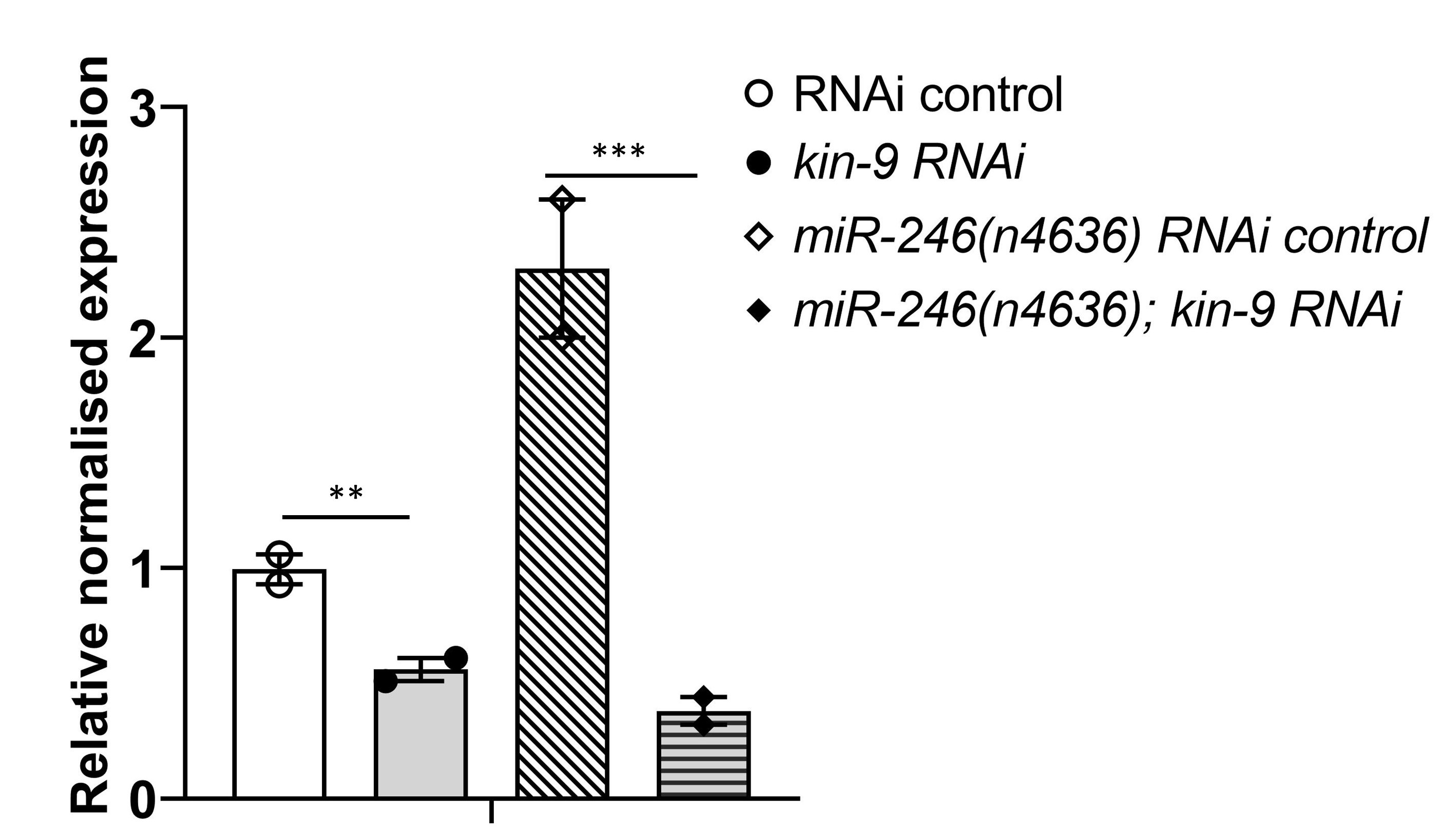

Supplement: Supplementary file 9 [file Image10.JPEG]

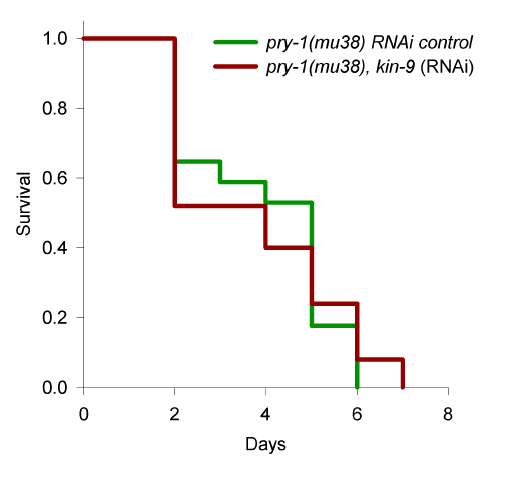

Supplement: Supplementary file 10 [file Image11.JPEG]

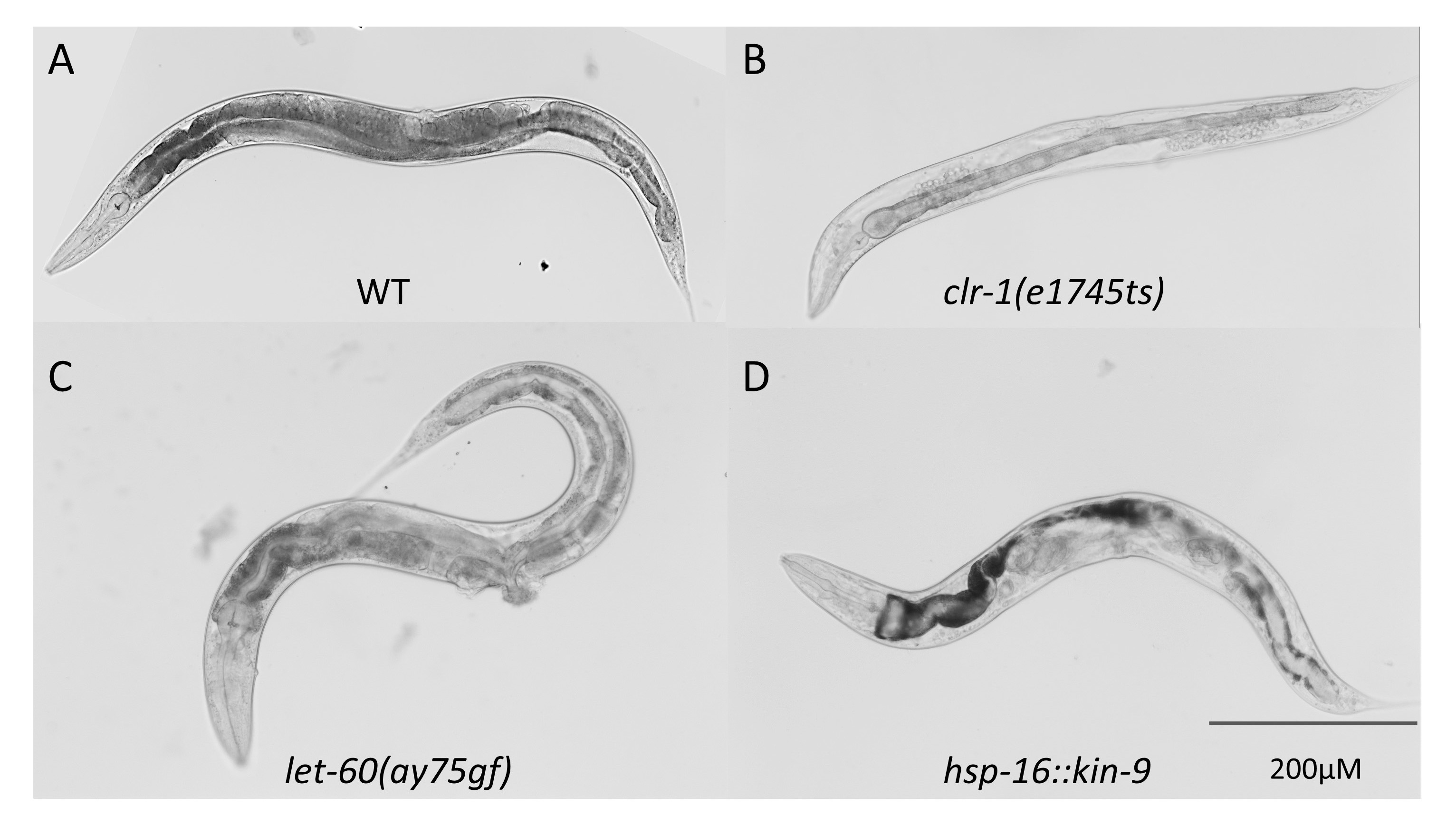

Supplement: Supplementary file 13 [file Image8.JPEG]

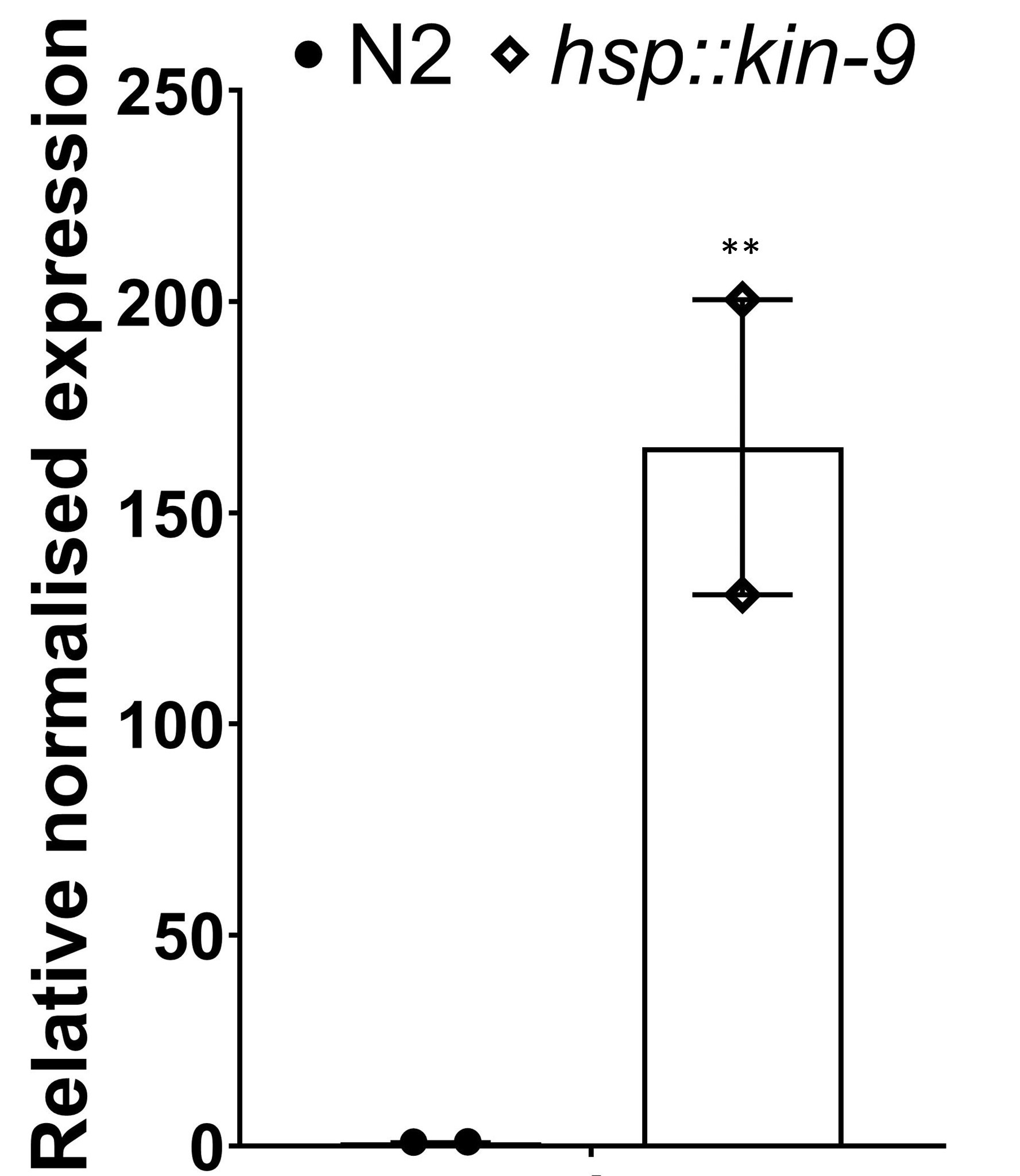

Supplement: Supplementary file 15 [file Image6.JPEG]
