# Supplementary material for: The FGFR4 Homolog KIN-9 Regulates Lifespan and Stress Responses in Caenorhabditis elegans
Source: Front Aging. 2022 May 20;3:866861. doi: 10.3389/fragi.2022.866861 (PMC9261393; doi:10.3389/fragi.2022.866861)
Supplement: Supplementary file 11 [file Table3.DOCX]

| **Growth temp** | **Genotype** | **RNAi treatments** | **Mean Lifespan (days)** | **Median Lifespan (days)** | **Maximum Lifespan (days)** | **N** | ***p* value** | **Figure** |
| --- | --- | --- | --- | --- | --- | --- | --- | --- |
| **15C** | **N2** |  | 21.3 ± 0.9 | 21 | 28 | 88 |  | S6A |
|  | ***kin-9(tm3973)*** |  | 22.5 ± 1.1 | 22 | 30 | 69 | ns |  |
| **20C** | **N2** |  | 15.7 ± 0.7 | 15 | 23 | 92 |  | 3A |
|  | ***kin-9(tm3973)*** |  | 17.7 ± 0.7 | 18 | 26 | 88 | < 0.001 |  |
| **25C** | **N2** |  | 14.5 ± 0.6 | 14 | 19 | 77 |  | S6B |
|  | ***kin-9(tm3973)*** |  | 16.2 ± 0.7 | 17 | 23 | 78 | < 0.001 |  |
| **20C** | **N2** | *empty vector* | 16.3 ± 0.4 | 16 | 22 | 38 |  | 3B |
|  |  | *kin-9* | 17.8 ± 0.5 | 19 | 24 | 48 | < 0.05 |  |
|  | **N2** | *empty vector* | 16.8 ± 0.5 | 16 | 22 | 61 |  | 4E |
|  |  | *kin-9* | 18.4 ± 0.6 | 19 | 24 | 67 | < 0.05 |  |
|  | ***miR-246(n3646)*** | *empty vector* | 10.9 ± 1.1 | 12 | 20 | 92 |  | 4E |
|  |  | *kin-9* | 14.7 ± 0.9 | 14 | 22 | 58 | < 0.01 |  |
|  | ***pry-1(mu38)*** | *empty vector* | 3.9 ± 0.4 | 4 | 6 | 52 |  | not shown |
|  |  | *kin-9* | 3.8 ± 0.4 | 4 | 6 | 64 | ns |  |
| **15C** | **N2** |  | 24.9 ± 0.9 | 26 | 30 | 43 |  | S6C |
|  | ***hsp::kin-9*** |  | 20.1 ± 0.6 | 20 | 28 | 35 | < 0.001 |  |
| **20C** | **N2** |  | 16.9 ± 0.9 | 16 | 23 | 48 |  | 3E |
|  | ***hsp::kin-9*** |  | 11.6 ± 0.6 | 10 | 21 | 45 | < 0.001 |  |
| **25C** | **N2** |  | 13.7 ± 0.4 | 14 | 16 | 54 |  | S6D |
|  | ***hsp::kin-9*** |  | 9.2 ± 0.6 | 10 | 16 | 35 | < 0.001 |  |
