# Supplementary material for: The FGFR4 Homolog KIN-9 Regulates Lifespan and Stress Responses in Caenorhabditis elegans
Source: Front Aging. 2022 May 20;3:866861. doi: 10.3389/fragi.2022.866861 (PMC9261393; doi:10.3389/fragi.2022.866861)
Supplement: Supplementary file 14 [file DataSheet1.DOCX]

**SUPPLEMENTARY DATA FILE 1**

**The FGFR4 homolog KIN-9 regulates lifespan and stress responses in *Caenorhabditis elegans***

**Avijit Mallick et al.**

**SUPPLEMENTARY MATERIALS AND METHODS**

***Strains***

**N2** *C. elegans* wild type, **CB3241** *clr-1(e1745)* *II*, **MT15020** *miR-246(n4636) IV***, NH2531** *let-60(ay75)/dpy-20(e1362) IV,* **DY662** *kin-9(tm3973) X,* **DY691** *unc-119(tm4063) III; bhEx289[pGLC158(kin-9p(4.3kb)::GFP)+unc-119(+)],* **DY692** *unc-119(tm4063) III; bhEx290[pGLC144(kin-9p(2.1kb)::GFP)+unc-119(+)],* **DY676** *bhEx285[pGLC146(hsp-16::kin-9) + pJH1774(myo-3p::wCherry)],* **DY700** *bhEx293[pGLC160(kin-9p(2.1kb)::GFP::kin-9 3’UTR)],* **DY703** *pry-1(mu38) I; bhEx293[pGLC160(kin-9p::GFP::kin-9 3’UTR)],* **DY705** *miR-246(n4636) IV; bhEx293[pGLC160(kin-9p::GFP::kin-9 3’UTR)].*

The *kin-9(tm3973)* deletion mutant was obtained from the National BioResource Project and was confirmed by sequencing (<https://shigen.nig.ac.jp/c.elegans>) (For primers used see **Supplementary Table 1**). The allele carries a 349 bp long deletion (flanking 25 nucleotides: TTTTGGAGTGCAACTAGTGGTCAAC and TCGCCTACCATCCTCATCTTGTGTC). The strain was outcrossed twice before performing experiments.

***Molecular Biology and transgenics***

RNA was extracted from synchronized L3 and day-1 adult animals. Protocols for RNA extraction, cDNA synthesis, and qPCR were described earlier. Briefly, total RNA was extracted using Trizol (Thermo Fisher, USA), cDNA was synthesized using the SensiFast cDNA synthesis kit (Bioline, USA), and qPCR was done using the SYBR green mix (Bio-Rad, Canada). Primers used for qPCR experiments are listed in **Supplementary Table 1**.

Plasmids were constructed as follows. To design pGLC144, a 2,182 bp PCR-amplified (using the primers GL1350 and GL1352) fragment, spanning the promoter region and a portion of the first exon of *kin-9b.1* and *kin-9c.1*, was subcloned into the vector pPD95.81, using SphI and SalI. For pGLC158, we cloned a 4,378 bp PCR-amplified (using the primers GL1431 and GL1432) fragment, spanning the promoter region and a portion of the first exon of the *kin-9a.1* gene, into the vector pPD95.81, using the restriction sites SalI and KpnI. The pGLC160 was derived from pGLC144. This was done by replacing the *unc-54* 3’ UTR in pGLC144 with the *kin-9* 3’ UTR using the restriction sites SpeI and EcoRI. The 554 bp of *kin-9* fragment was obtained by PCR (using the primers GL1497 and GL1498). For pGLC146, we cloned a 4,715 bp PCR-amplified (using the primers GL1353 and GL1354) fragment, spanning the full-length coding sequence of the *kin-9* gene, into the vector pPD49.83, using the restriction sites KpnI and SacI.

To generate the DY691 and DY692 transgenic lines, *unc-119(tm4063)* mutants were injected with pGLC158 and pGLC144 plasmids (50 ng/µL) respectively, along with the rescue plasmid unc-119(+) (40 ng/µL). DY700 strain was generated by injecting 50 ng/µL of the pGLC160 plasmid in the N2 background. DY676 strain was generated by injecting 20 ng/µL of the pGLC146 plasmid with 30 ng/µL of the coinjection marker pJH1774 (*myo-3::wCherry*) in the N2 background.

***Fluorescent microscopy***

Animals were paralyzed in 10mM Sodium Azide and mounted on glass slides with 2% agar pads and covered with glass coverslips for immediate image acquisition using Zeiss Apotome microscope and software.

***Body bending and pharyngeal pumping***

The rate of body bending per 1min and the rate of pharyngeal pumping per 30sec for adults were analyzed over the period of 4 days. Hermaphrodites were analyzed for these phenotypes under the dissecting microscope in isolation on OP50 plates. Pharyngeal pumping was assessed by observing the number of pharyngeal contractions for 30sec. For body bending assessment, animals were stimulated by tapping once on the tail of the worm using the platinum wire pick where one body bend corresponded to one complete sinusoidal wave of the worm. Only animals that moved throughout the duration of 1 min were included in the analysis.

Body movement defects of *clr-1* mutants were quantified on a 0-3 scale: no movement (0), very little movement (1), slow movement (2) and healthy movement (3). For this experiment, animals were grown at 20°C till L4 stage on RNAi plates. They were then placed at 25°C till adulthood to examine body movement.

***Stress assay***

Oxidative (paraquat) and endoplasmic reticulum mediated stress (tunicamycin) stress experiments were performed using 200mM paraquat (PQ) (Thermo Fisher Scientific, USA) and 25ng/μL tunicamycin (Sigma-Aldrich, Canada) respectively. Animals were incubated, for 2hrs or over a period of 6hrs, following the previously published protocol. All the final working concentrations were made in M9 instead of water. At least 50 animals were tested for each strain in each replicate. Heat stress experiments were performed by incubating the NGM plate containing at least 50 adult animals at 35°C for either 2hrs or over a period of 12hrs. Mean and standard deviation was determined from experiments performed in duplicate. Animals were considered dead if they had no response following a touch using the platinum wire pick and showed no thrashing or swimming movement in M9. Moreover, dead animals usually had an uncurled and straight body shape in comparison to the normal sinusoidal shape of worms.

***Statistical analyses***

Statistics analyses were performed using GraphPad prism 9, SigmaPlot software 11, CFX Maestro 3.1, and Microsoft Office Excel 2019. For lifespan data, survival curves were estimated using the Kaplan- Meier test, and differences among groups were assessed using the log-rank test. qPCR data were analyzed using Bio-Rad CFX Maestro 3.1 software. For all other assays, data from repeat experiments were pooled and analyzed together and statistical analyses were done using GraphPad Prism 9. p values less than 0.05 were considered statistically significant.
